# Supplementary material for: Environment and Local Substrate Availability Effects on Harem Formation in a Polygynous Bark Beetle
Source: Insects. 2021 Jan 24;12(2):98. doi: 10.3390/insects12020098 (PMC7912661; doi:10.3390/insects12020098)
Supplement: Supplementary file 1 [file insects-12-00098-s001.pdf]

SUPPLEMENTARY MATERIAL

Table S1. Top generalised linear mixed models identified using model selection with AIC_C values for predicting the harem size (females per male) of *Ips grandicollis* bark beetles. The random effect was the log ID number. Marginal R^2 = 0.014, conditional R^2 = 0.014.

Model components	DF	AIC_c	ΔAIC_c	w_i
**HS ~ Enviro**	3	8508.4	0	0.393
**HS ~ Enviro + TSA + Enviro* TSA**	5	8509.9	1.6	0.180
**HS ~ Resources + Enviro**	4	8510.4	2.0	0.144
**HS ~ Enviro + DNH + Enviro * DNH**	5	8511.2	2.8	0.096
**HS ~ Resources + Enviro +TSA**	5	8511.3	2.9	0.056
**HS ~ Resources + Enviro + Resources * Enviro**	5	8512.3	3.9	0.039
**HS ~ Resources + Enviro +TSA + DNH**	6	8513.0	4.6	<0.0001
**HS ~ Resources + TSA + Resources * TSA**	5	8532.7	24.3	<0.0001
**HS ~ TSA**	3	8534.9	26.5	<0.0001
**Null model**	2	8535.5	27.1	<0.0001
**HS ~ DNH**	3	8537.1	28.7	<0.0001
**HS ~ Resources**	3	8537.4	29.1	<0.0001
**HS ~ DNH + TSA + DNH * TSA**	5	8537.9	29.6	<0.0001
**HS ~ Resources + DNH + Resources * DNH**	5	8540.8	32.3	<0.0001

HS = Harem size, *Enviro* = environment (harvested or unharvested plantation), *Resources* = resource size (10 logs or 5 logs), *TSA* = total surface area of log (m^2), *DNH* = distance to the nearest harem.

Table S2. Model selection using AIC_C values for GLMM predicting the total number of Ips grandicollis bark beetles per log. The random effect was the tree ID number (the tree the logs were cut from). Marginal R^2 = 0.115, conditional R^2 = 0.267.

**Model components**	**DF**	**AIC_c**	**ΔAIC_c**	**w_i**
**Total ~ Enviro * Resources + TSA + Enviro + Resources**	6	1877.34	0	0.8504
**Total ~ Resources + TSA**	4	1881.98	4.6379	0.0837
**Total ~ Enviro + Resources + TSA**	5	1882.45	5.1124	0.0660
**Total~Enviro * Resources + Enviro + Resources**	5	1930.05	52.7113	<0.0001
**Total ~ Resources**	3	1937.66	60.3155	<0.0001
**Total ~ Enviro + Resources**	4	1938.38	61.0337	<0.0001
**Total ~ TSA**	3	1942.11	64.7712	<0.0001
**Total ~ Enviro + TSA**	4	1943.16	65.8149	<0.0001
**Null model**	2	1991.30	113.8237	<0.0001
**Total ~ Enviro**	3	1992.30	114.9605	<0.0001

Total = sum of males and females on each log, *Enviro* = environment (harvested or unharvested plantation), *Resources* = resource size (10 logs or 5 logs), *TSA* = total surface area of logs (m^2).

Table S3. Model selection using AIC_C values for GLMM predicting the distance to the nearest harem (male nuptial chamber). The random effect was the log ID number.

Model components	DF	AIC_c	ΔAIC_C	w_i
**DNH ~ Resources**	4	-10078.6	0	0.9551
**DNH ~ Resources + TSA**	5	-10072.4	6.2054	0.0429
**DNH ~ Resources + Enviro**	5	-10066.2	12.4643	0.0018
**DNH ~ Resources + Enviro + TSA**	6	-10059.7	18.9248	<0.0001
**DNH ~ Resources + Enviro + TSA + Enviro * Resources**	6	-10054.1	24.5480	<0.0001
**Null model**	3	-10051.1	27.4934	<0.0001
**DNH ~ Resources + Enviro**	7	-10047.6	30.9945	<0.0001
**DNH ~ TSA**	4	-10045.7	32.8986	<0.0001
**DNH ~ Enviro**	4	-10038.2	40.3955	<0.0001
**DNH ~ Enviro + TSA**	5	-10032.6	46.0244	<0.0001

DNH = distance to the nearest harem, *Enviro* = environment (harvested or unharvested plantation), *Resources* = resource size (10 logs or 5 logs), *TSA* = total surface area of log (m^2).

Table S4. Model selection using AIC_C values for GLMM predicting the density of males on each log (males per m^2). The random effect was the replicate (i.e. the log pile number).

Model components	DF	AIC_C	ΔAIC_C	w_i
**Density ~ Resources + Enviro + Resources * Enviro**	6	1347.77	0	0.9562
**Density ~ Resources + Enviro**	5	1354.29	6.5224	0.0367
**Density ~ Resources**	4	1357.57	9.8045	0.0071
**Density ~ Enviro**	4	1370.89	23.1157	<0.0001
**Null model**	3	1374.20	26.4271	<0.0001

Density = males per m^2, *Enviro* = environment (harvested or unharvested plantation), *Resources* = resource size (10 logs or 5 logs).

Table S5. Mean proximity index (PI) of females in each harem size in harvested and unharvested environments.

Number of females	Environment	N	Mean PI	p
**3**	Harvested	319	0.1974	0.3954
	Unharvested	410	0.1822	0.3708
**4**	Harvested	483	0.2255	0.3574
	Unharvested	348	0.2055	0.3056
**5**	Harvested	211	0.2098	0.2194
	Unharvested	132	0.2142	0.2481
**6**	Harvested	25	0.2059	0.2188
	Unharvested	33	0.1653	0.1204
**7**	Harvested	3	0.2288	0.2864
	Unharvested	6	0.1728	0.1415

Table S6. Mean proximity index (PI) of females in each harem size in high (10 logs) and low (5 logs) experimental resource availability.

Number of females	Resources	N	Mean PI	p
**3**	5 logs	268	0.199	0.3973
	10 logs	458	0.1829	0.3702
**4**	5 logs	325	0.2102	0.3234
	10 logs	506	0.2216	0.3534
**5**	5 logs	121	0.1983	0.1899
	10 logs	222	0.2187	0.2575
**6**	5 logs	17	0.2781	0.4858
	10 logs	41	0.2056	0.2190
**7**	5 logs	4	0.2307	0.2889
	10 logs	5	0.1424	0.0653

Table S7. Mean minimum distance between two females in each harem size in harvested and unharvested environments.

Number of females	Environment	N	Mean min distance	p
**2**	Harvested	128	3.33	0.7498
	Unharvested	255	3.54	0.7500
**3**	Harvested	319	2.22	0.6205
	Unharvested	410	2.30	0.6205
**4**	Harvested	483	1.71	0.5709
	Unharvested	348	1.79	0.5710
**5**	Harvested	211	1.29	0.4079
	Unharvested	132	1.30	0.4080
**6**	Harvested	25	1.06	0.4646
	Unharvested	33	1.01	0.4646
**7**	Harvested	3	0.60	0.4744
	Unharvested	6	0.78	0.4745

Table S8. Mean minimum distance between two females in each harem size in high (10 logs) and low (5 logs) experimental resource availability.

Number of females	Resources	N	Mean PI	p
**2**	5 logs	140	3.39	0.7498
	10 logs	243	3.51	0.7499
**3**	5 logs	268	2.26	0.6206
	10 logs	458	2.27	0.6206
**4**	5 logs	325	1.78	0.5710
	10 logs	506	1.72	0.5709
**5**	5 logs	121	1.30	0.4080
	10 logs	222	1.28	0.4079
**6**	5 logs	17	1.05	0.4646
	10 logs	41	1.02	0.446
**7**	5 logs	4	0.65	0.4744
	10 logs	5	0.78	0.4745
